# Intentional overdose with insulin: prognostic factors and toxicokinetic/toxicodynamic profiles

**DOI:** 10.1186/cc6168

**Published:** 2007-10-28

**Authors:** Bruno Mégarbane, Nicolas Deye, Vanessa Bloch, Romain Sonneville, Corinne Collet, Jean-Marie Launay, Frédéric J Baud

**Affiliations:** 1 Assistance Publique – Hôpitaux de Paris, Hôpital Lariboisière, Réanimation Médicale et Toxicologique, INSERM U705, CNRS, UMR 7157, Université Paris 7, Université Paris 5, 2 Rue Ambroise Paré, 75010, Paris, France; 2 Assistance Publique – Hôpitaux de Paris, Hôpital Lariboisière, Laboratoire de Biochimie et de Biologie Moléculaire, 2 Rue Ambroise Paré, 75010, Paris, France

## Abstract

**Introduction:**

Prognostic factors in intentional insulin self-poisoning and the significance of plasma insulin levels are unclear. We therefore conducted this study to investigate prognostic factors in insulin poisoning, in relation to the value of plasma insulin concentration.

**Methods:**

We conducted a prospective study, and used logistic regression to explore prognostic factors and modelling to investigate toxicokinetic/toxicodynamic relationships.

**Results:**

Twenty-five patients (14 female and 11 male; median [25th to 75th percentiles] age 46 [36 to 58] years) were included. On presentation, the Glasgow Coma Scale score was 9 (4 to 14) and the capillary glucose concentration was 1.4 (1.1 to 2.3) mmol/l. The plasma insulin concentration was 197 (161 to 1,566) mIU/l and the cumulative amount of glucose infused was 301 (184 to 1,056) g. Four patients developed sequelae resulting in two deaths. Delay to therapy in excess of 6 hours (odds ratio 60.0, 95% confidence interval 2.9 to 1,236.7) and ventilation for longer than 48 hours (odds ratio 28.5, 95% confidence interval 1.9 to 420.6) were identified as independent prognostic factors. Toxicokinetic/toxicodynamic relationships between glucose infusion rates and insulin concentrations fit the maximum measured glucose infusion rate (E_max_) model (E_max _29.5 [17.5 to 41.1] g/hour, concentration associated with the half-maximum glucose infusion rate [EC_50_] 46 [35 to 161] mIU/l, and R^2 ^range 0.70 to 0.98; *n *= 6).

**Conclusion:**

Intentional insulin overdose is rare. Assessment of prognosis relies on clinical findings. The observed plasma insulin EC_50 _is 46 mIU/l.

## Introduction

Contrasting with the common occurrence of insulin-induced hypoglycaemia in type 1 diabetes patients, deliberate overdose with insulin are rarely reported [1]. In the 2005 Annual Report of the American Association of Poison Control Centers, only 3,934 out of the 2,424,180 reported exposures to substances involved insulin [2]. Consistent with this, a recent study in a poison centre [3] estimated the annual rate of enquiries secondary to insulin overdose at 20. In a series of diabetic poisoned patients, fewer than 5% of suicide attempts involved insulin [4]. Similarly, in a series of nondiabetic poisoned patients presenting with toxic hypoglycaemia, fewer than 1% had self-injected insulin [5].

Deliberate self-poisoning with insulin may result in severe symptoms, including hypoglycaemic coma, neurological impairment and death [1,6]. The major difference between insulin therapeutic mistake and deliberate overdose is the much greater dose of insulin used in the latter, leading to elevated and prolonged need for glucose. Prognostic factors in insulin overdose remain subject to debate, and the optimal modalities of glucose therapy are not known. It is still unknown whether the necessary rate of glucose infusion may be predicted by determining the plasma insulin level. We therefore conducted the presented study with the following goals: to describe patients admitted to the intensive care unit (ICU) for severe insulin poisoning; to investigate prognostic factors in insulin overdose; and to determine the association between rate of glucose infusion and plasma insulin concentration, by examining toxicokinetic/toxicodynamic (TK/TD) relationships.

## Materials and methods

### Descriptive study setting

We prospectively reviewed the charts of all consecutive patients admitted to our ICU from January 1999 to December 2005 because of intentional insulin overdose. The circumstances of poisoning, clinical presentation, and results of capillary glucose concentrations, routine blood tests and toxicological screening were recorded. Data regarding clinical features and glucose levels were obtained at the scene and on ICU admission.

All the patients were managed in accordance with the standard treatment guidelines that are currently used in our department. Glucose infusion rate was continuously adapted based on hourly determination of capillary glucose to maintain a blood glucose level in the range of 10 to 12 mmol/l. Systematic attempts were made to reduce the infusion rate, but the rate was returned to the previous level if evidence of hypoglycaemia was detected. We calculated the cumulative amount of glucose given orally and intravenously to each patient until the time point at which the effects of injected insulin were deemed to have ceased. This time was determined, as previously proposed [7], from therapy initiation to the time point at which no further hypoglycaemic episodes occurred along with one of the following events: discontinuation of the intravenous line; decrease in intravenous glucose infusion to under 2.5 g/hour or change to a nonglucose solution; insulin restart if the patient was type 1 diabetic; or measurements of a glucose concentration above 6.5 mmol/l on two occasions or more than 8.25 mmol/l once. Physiological variables measured on admission were used to calculate the Simplified Acute Physiology Score (SAPS) II [8]. At ICU discharge, the Glasgow-Pittsburgh Cerebral Performance Category (CPC) was determined [9]. For data analysis, the patient population was split into two groups according to the following outcomes: 'favourable' (defined as CPC 1 or 2) and 'unfavourable' (defined as CPC 3 to 5). Unfavourable outcomes include death and severe neurological impairment on ICU discharge.

### Analysis of TK/TD relationships

We conducted a TK/TD analysis between the glucose infusion rate in order to normalize the capillary glucose concentration (as a toxicodynamic parameter) and the corresponding serum insulin concentrations (as a toxicokinetic parameter). This study was approved by our institutional ethics committee, and verbal informed consent was obtained from the patient when conscious or from the next of kin when not.

Plasma insulin and C-peptide concentrations were determined using the same samples as those used for glucose measurements. Plasma insulin concentration was measured using a commercial Microparticle Enzyme Immunoassay (MEIA technology, Axsym system; Abbott Japan Co., Ltd, Osaka, Japan; limit of quantification 1.0 mU/l). Plasma C-peptide concentration was determined with a solid-phase competitive chemiluminescent enzyme immunoassay (Immulite; Diagnostic Products Corporation, Los Angeles, CA, USA; limit of quantification 0.5 ng/ml). Venous blood samples were obtained at the discretion of the attending physicians. The rate of glucose infusion was prospectively recorded at each blood sampling, and nurses in charge were blinded to the results of plasma insulin measurement. For each value of plasma insulin concentration measured at time t_n _(with t_0 _being the time of initial therapy and t_1 _being time of the first plasma insulin measurement), we attributed a value of glucose infusion rate obtained by dividing the quantity of glucose administered from (t_n-1 _+ t_n_)/2 to (t_n+1 _+ t_n_)/2 by the delay (t_n+1 _- t_n-1_)/2. For the first value at time t_1_, the glucose infusion rate was obtained by dividing the quantity of glucose administered from t_0 _to (t_1 _+ t_2_)/2 by the corresponding time. Regarding the toxicokinetic study, we considered all plasma insulin values in type 1 diabetic patients, provided that no insulin was re-administrated. In nondiabetic and type 2 diabetic patients, we only considered plasma insulin concentrations above 20 mU/l (the upper limit of normal in the fasting state), provided that their corresponding plasma C-peptide concentration was under 0.5 ng/ml. The half-time of the disappearance rate of exogenous insulin was calculated using the method proposed by Pearson and coworkers [10]. Studies of toxicokinetic (noncompartmental analysis) and TK/TD relationships were performed using a computerized curve fitting program (Win-Nonlin Pro 4.1; Pharsight, Mountain View, CA, USA).

### Statistical analysis

Results are expressed as median (25th to 75th percentiles) or percentage when appropriate. Fisher's exact tests and nonparametric tests were used for between-group comparisons. Correlations were quantified using Pearson's linear correlation coefficient. A stepwise logistic regression was used to explore the effects of several variables on the outcome (considering death or significant neurological sequelae at ICU discharge to represent an unfavourable outcome) and the duration of ICU stay (considering an ICU stay >10 days to represent an unfavourable outcome). The predicted proportion of unfavorable outcomes was assumed to follow the logistic model. The step selection was based on the maximum likelihood ratio. Odds ratio (OR) were calculated along with 95% confidence interval (CI). *P *< 0.05 was considered statistically significant.

## Results

### Descriptive analysis and study of prognostic factors

Over a 6-year period, 25 patients (14 females and 11 male, age 46 [36 to 58] years and SAPS II score 25 [19 to 51]) were admitted in our ICU because of intentional insulin poisoning. A past psychiatric history was noted in 20 of the 25 patients (80%) and diabetes mellitus in 13 of the 25 patients (52%). The five nondiabetic patients (20%) were nurses. Rapid-acting insulin (injected amount 300 [138 to 525] IU) was involved in 14 out of 25 patients, while intermediate-acting or slow-acting insulin (300 [170 to 1,300] IU) was used by 13 out of 25 patients. Two patients self-injected both insulin types. Drug ingestion, mainly benzodiazepines, was also identified in 68% of patients. The interval between insulin self-injection and pre-hospital glucose administration was 2.7 (1 to 5) hours. At presentation, Glasgow Coma Scale score was 9 (4 to 14), systolic blood pressure was 120 (110 to 158) mmHg, pulse rate was 95 (80 to 111) beats/minute and respiratory rate was 20 (18 to 28) breaths/minute. The temperature was 36.0°C (35.0°C to 37.0°C). At the scene, the capillary glucose concentration was 1.4 (1.1 to 2.3) mmol/l. Six patients were mechanically ventilated for persistent coma despite correction of hypoglycaemia. On ICU admission, the blood glucose was 5.3 (2.8 to 7.3) mmol/l, plasma potassium was 3.3 (3.0 to 3.8) mmol/l, plasma lactate 2.0 (1.7 to 2.8) mmol/l, and the maximal observed plasma insulin concentration was 197 (161 to 1,566) IU/ml.

All patients received an infusion of 30% dextrose in water titrated to blood glucose levels. Six patients received additional 50% dextrose in water and five glucagon injections. The total amount of infused glucose was 301 (184 to 1,056) g. The total duration of glucose infusion was 32 (12 to 68) hours. In the ICU, seven patients (28%) were mechanically ventilated (duration 15 [3 to 51] days) and five (20%) received catecholamine infusions for circulatory failure. Four patients (16%) developed an aspiration pneumonia. Two patients developed an acute respiratory distress syndrome confirmed by pulmonary wedge pressure measurements.

**Figure 1:**
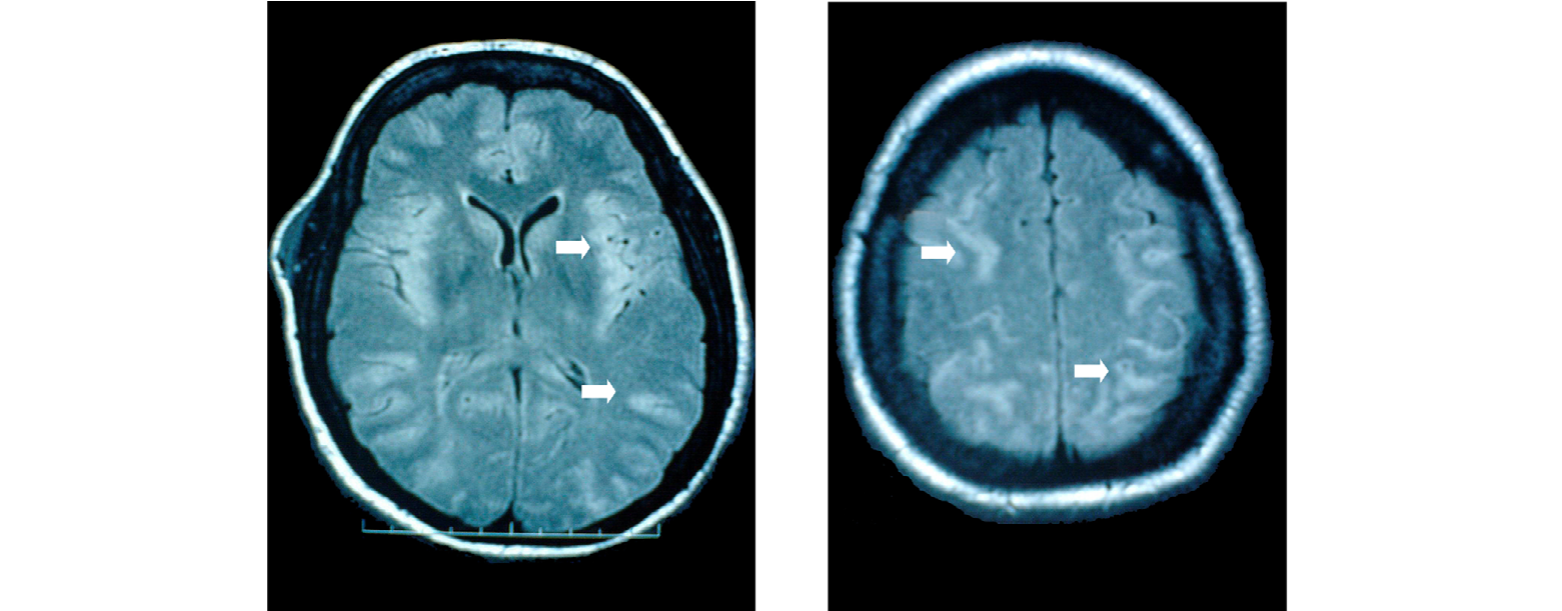
MRI findings in hypoglycemia-related encephalopathy. Cerebral fast fluid-attenuated inversion recovery magnetic resonance imaging (MRI) in a patient suffering from a severe hypoglycaemia-related encephalopathy on day 3 after deliberate insulin self-poisoning. The disseminated hypersignals of the cerebral gray matter (plain arrows) disappeared on day 30, whereas neurological impairments persisted.

Final outcome was favourable in 21 out of 25 patients (Table 1). Two patients died in the ICU, following withholding and withdrawal of life-sustaining treatments because of severe hypoglycaemic encephalopathy, associated in one case with a terminal phase cancer. Two other patients suffered from significant neurological sequelae at ICU discharge (CPC 3), including cognitive and memory impairments. In the patient who died on day 85 with a severe hypoglycaemia-related encephalopathy, fast fluid-attenuated inversion recovery magnetic resonance imaging showed disseminated hypersignals in the cerebral gray matter at day 3 (Figure 1). Interestingly, all these signal abnormalities disappeared on day 30, whereas marked cerebral atrophy was observed and neurological disabilities persisted. A stepwise multiple regression logistic regression model showed that a delay between insulin injection and first medical treatment in excess of 6 hours (OR 60.0, 95% CI 2.9 to 1,236.7) and a duration of mechanical ventilation in excess of 48 hours (OR 28.5, 95% CI 1.9 to 420.6) appeared to be significant independent predictors of unfavourable outcome of insulin poisoning.

**Table 1:** Comparison of patient clinical parameters according to the outcome

Parameter	Favourable outcome (*n *= 21)	Unfavourable outcome (*n *= 4)	*P*
Age (years)	46 (36 to 58)	45 (26 to 67)	0.9
SAPS II	23 (18 to 36)	62 (61 to 69)	0.002
Total amount of injected insulin (IU)	250 (135 to 988)	450 (250 to 600)	0.6
Delay before pre-hospital management (hours; *n *= 22)	2 (0.9 to 3.4)	9 (7.5 to 9.8)	0.009
On the scene			
Glasgow Coma Scale score	12 (8 to 14)	4 (4 to 6)	0.06
Capillary glucose level (mmol/l)	1.4 (1.1 to 2.3)	0.7 (1.5 to 4.1)	0.9
Mechanical ventilation (%)	14	75	0.03
On ICU admission			
GCS score before dextrose administration	15 (14 to 15)	6 (4 to 10)	0.003
Systolic blood pressure (mmHg)	120 (105 to 158)	125 (112 to 158)	0.7
Pulse rate (beats/min)	81 (74 to 101)	126 (112 to 143)	0.005
Temperature (°C)	35.6 (35.0 to 36.6)	36.9 (36.7 to 37.1)	0.04
Blood glucose level (mmol/l)	6.2 (4.0 to 8.0)	2.3 (1.0 to 3.5)	0.03
Maximum plasma insulin concentration (IU/l; *n *= 15)	192 (153 to 1,853)	209 (ND)	0.8
Plasma lactate concentration (mmol/l)	2.0 (1.7 to 2.8)	2.0 (1.8 to 2.9)	0.8
Mechanical ventilation in ICU (%)	19	75	0.05
Amount of infused glucose (g)	282 (167 to 1,056)	886 (574 to 1,410)	0.2
Duration of glucose infusion (hours)	24 (12 to 62)	57 (31 to 69)	0.4
Duration of ICU stay (days)	3 (2 to 6)	42 (5 to 82)	0.04

The patients were classified according to their Glasgow-Pittsburgh Cerebral Performance Category (CPC) on intensive care unit (ICU) discharge in two outcome groups: 'favourable' (CPC 1 or 2) and 'unfavourable' (CPC 3 to 5). Values are expressed as median (25th to 75th percentile). GCS, Glasgow Coma Scale; SAPS, Simplified Acute Physiology Score II.

**Figure 2:**
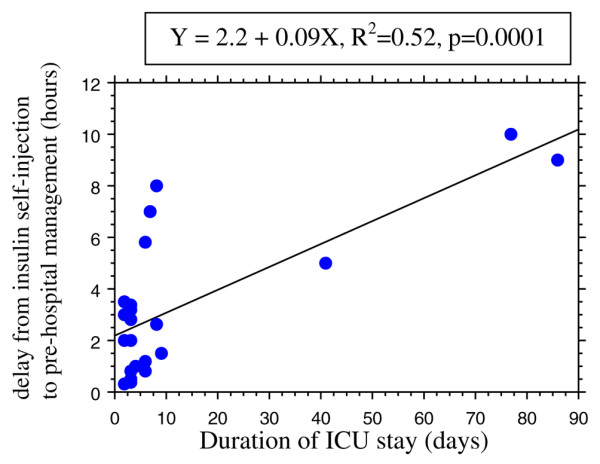
Delay from insulin self-injection to pre-hospital management versus duration of ICU stay. Shown is the correlation between the delay from insulin self-injection to pre-hospital management and the duration of intensive care unit (ICU) stay in 22 cases of insulin self-poisoning.

**Figure 3:**
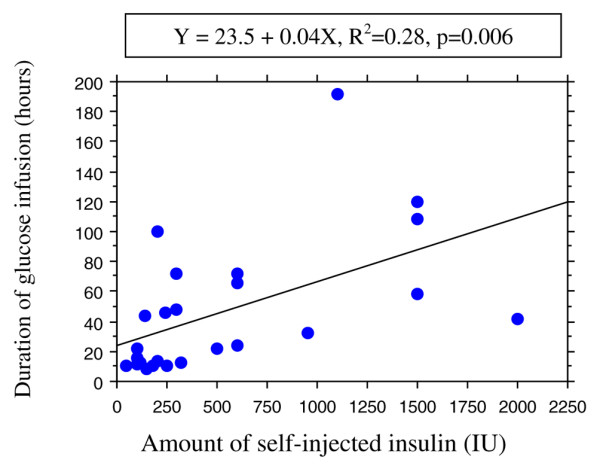
Duration of glucose infusion versus self-injected insulin dose. Shown is the correlation between the duration of glucose infusion and the self-injected insulin dose in 25 cases of insulin self-poisoning.

There was no significant correlation between plasma insulin level and the amount of injected insulin (R^2 ^= 0.07, *P *= 0.9; *n *= 15). There was a significant correlation between the duration of ICU stay and the delay to initial therapy (R^2 ^= 0.52, *P *= 0.0001; *n *= 22; Figure 2). There was no significant correlation between the amount of administered glucose and the amount of injected insulin (R^2 ^= 0.12, *P *= 0.1; *n *= 25). A weak correlation was found between the duration of glucose infusion and the self-injected insulin amount (R^2 ^= 0.28, *P *= 0.006; *n *= 25; Figure 3).

The duration of ICU stay was 3 (3 to 7) days. Comparisons using univariate analysis showed that the following factors differed significantly according to length of ICU stay (≤ days versus > 10 days): age (*P *= 0.001), SAPS II score (*P *< 0.001), amount of injected insulin (*P *< 0.001), interval between insulin injection and first medical treatment (*P *< 0.001), initial capillary glucose concentration (*P *= 0.003), initial Glasgow Coma Scale score (*P *= 0.03), mechanical ventilation requirement (*P *= 0.03), maximum observed plasma insulin level (*P *< 0.001), cumulative amount of administered dextrose (*P *< 0.001) and onset of sequelae (*P *= 0.04). A stepwise multiple regression logistic regression model showed that SAPS II score above 40 (OR 123.8, 95% CI 1.0 to 157.2) and occurrence of severe hypoglycaemic encephalopathy (CPC 3 to 5; OR 20.0, 95% CI 1.2 to 331.0) were significant independent predictors of ICU stay longer than 10 days.

### Study of insulin kinetics and toxicokinetic/toxicodynamic relationships

**Figure 4:**
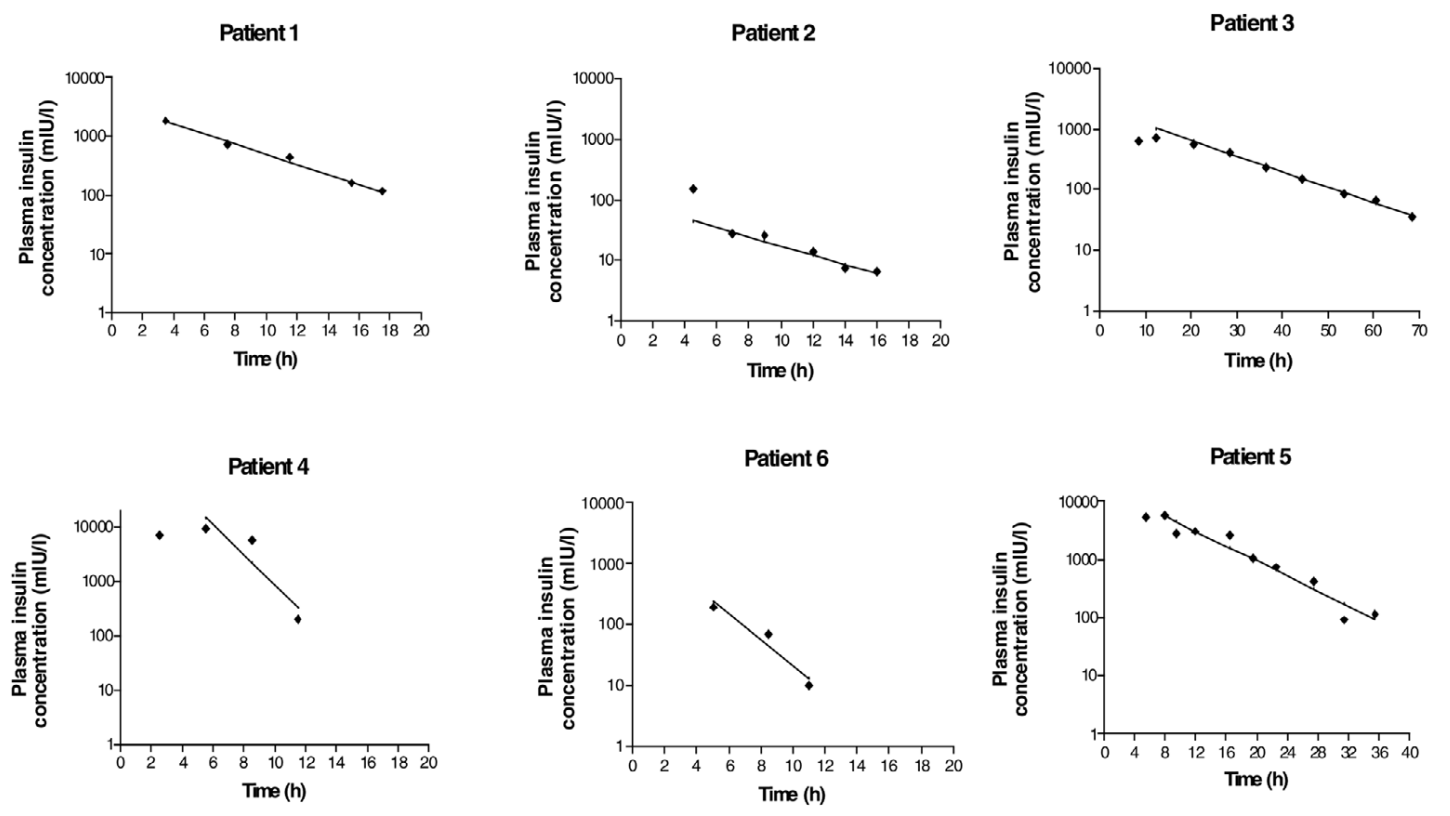
Plasma toxicokinetics of insulin in six severely insulin-poisoned patients.

**Figure 5:**
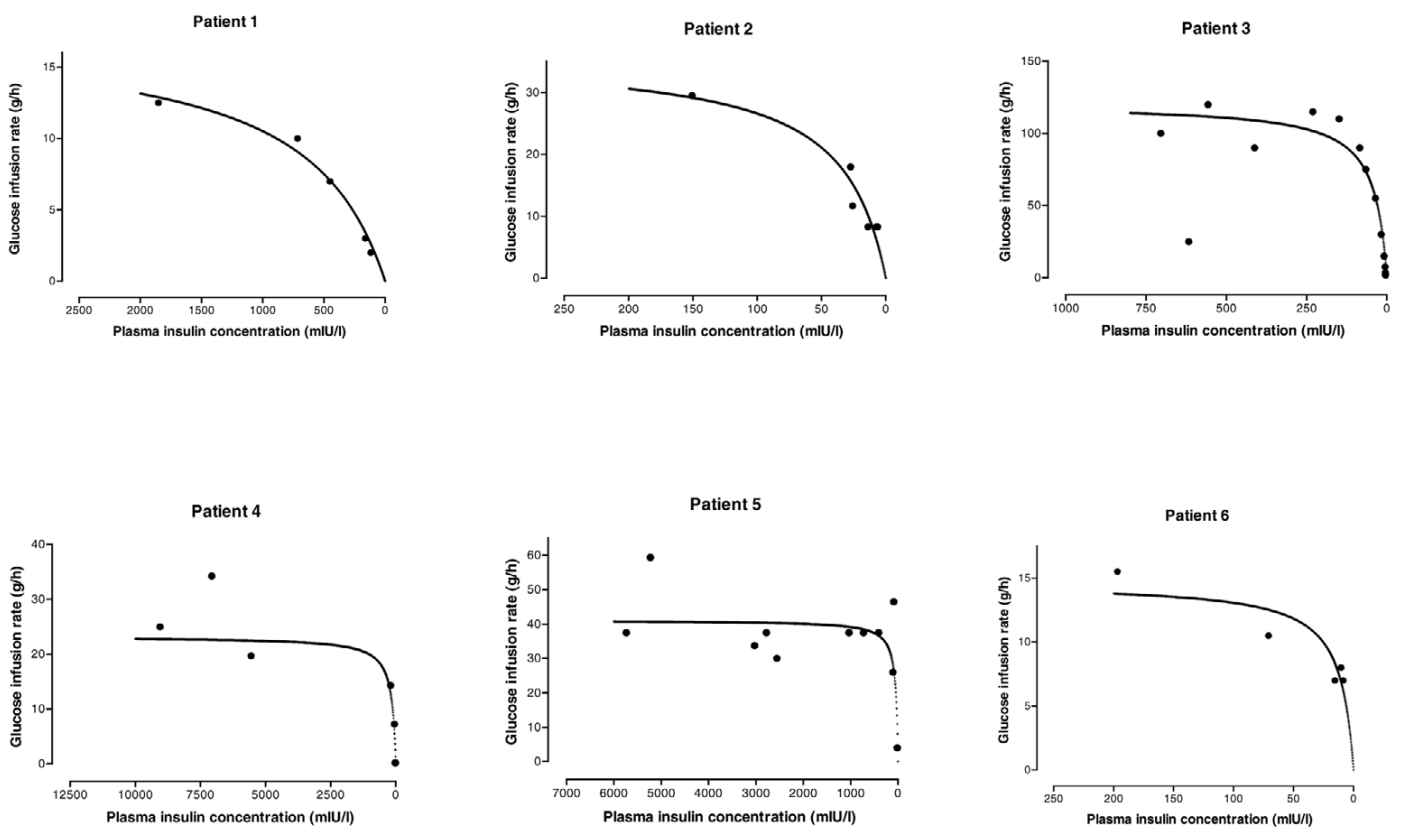
TK/TD relation between glucose infusion rate and plasma insulin concentrations. Shown are the toxicokinetic/toxicodynamic (TK/TD) relationships between glucose infusion rate and plasma insulin concentrations in six acutely insulin-poisoned patients.

Kinetics of insulin and TK/TD relationships were conducted in six patients, including three nondiabetic patients, two type 1 diabetic patients and one type 2 diabetic patient (Table 2). The decrease in exogenous insulin concentrations using a semi-logarithmic scale was linear, exhibiting first-order kinetics (Figure 4). The terminal half-life was 3.8 (1.5 to 4.6) hours. During the course of poisoning, TK/TD relationships between the glucose infusion rate (E) and insulin concentrations (C) fit the E_max _model E = (E_max _× C)/(EC_50 _+ C), where E_max _is the maximum measured glucose infusion rate and EC_50 _is the concentration associated with the half-maximum glucose infusion rate (Figure 5). In these six patients the maximal observed plasma insulin concentration C_max _was 1,279 (197 to 5,740) mIU/l, the E_max _was 29.5 (17.5 to 41.1) g/hour and the EC_50 _was 46 (35 to 161) mIU/l (Table 2).

**Table 2:** Characteristics and parameters of TK/TD relationships regarding the rate of glucose infusion versus insulin concentrations in six deliberate insulin intoxications

Patient	Sex/age (years)	Diabetes	Insulin type/dose	Delay to initial therapy (hours)	Lowest blood glucose level (mmol/l)	E_max _(g/h)	EC_50 _(mU/l)	C_max _(mIU/l)	R^2^	T_1/2 _(hours)	Outcome
1	Male/60	Type 1	Rapid-acting/500 IU	2.0	0.8	17.5	667	1,853	0.98	3.5	Alive
2	Female/40	Type 1	30% rapid-acting/70% slow-acting/120 IU	0.8	0.8	36	35	151	0.91	4.0	Alive
3	Female/52	Type 2	Slow-acting/1,500 IU	2.6	2.0	119.9	42	704	0.71	11.7	Alive
4	Female/45	Nondiabetic	Rapid-acting/100 IU	0.3	1.1	23.1	161	9,053	0.88	0.8	Alive
5	Male/16	Nondiabetic	Slow-acting/1,500 IU	1.5	2.5	41.1	50	5,740	0.70	4.6	Alive
6	Female/60	Nondiabetic	Rapid-acting/1,200 IU	1.0	1.7	14.6	12	197	0.79	1.5	Alive

C_max_, maximal observed concentration; EC_50_, concentration associated with half-maximal effect; E_max_, maximum possible measured glucose infusion rate; R^2^, correlation coefficient for the TK/TD model; T_1/2_, half-life; TK/TD, toxicokinetic/toxicodynamic.

## Discussion

Despite the widespread use of insulin, overdoses are infrequently reported. In comparison, sulfonylureas are the most frequently identified antidiabetic agent in human poisonings [11]. Insulin causes the greatest number of major and serious problems, whereas biguanides lead to most deaths. In our study, which included 25 patients admitted to our ICU because of severe insulin self-poisoning, four patients developed significant sequelae that resulted in two deaths. Consistent with this, in a large study assessing outcomes following 160 enquiries regarding insulin overdose recorded in a regional poison unit [3], full recovery occurred in 94.7% of patients while 2.7% patients had cerebral defects and 2.7% died. Hypoglycaemic encephalopathy is the most feared consequence of self-poisoning with insulin. The cortex, caudate, putamen and hippocampus are considered to be most vulnerable to hypoglycaemia. Selective regional brain vulnerability is related to differences in glucose content, glucose influx, amino acid distribution and inhibition of cerebral protein synthesis. Diffusion-weighted magnetic resonance imaging is therefore an excellent tool for evaluating patients who have self-poisoned with insulin, because it has the ability to detect cytotoxic damage early and can demonstrate (as in one of our patients) heterogeneous high intensity areas in both cortex and subcortex [12].

### Prognosis of severe acute insulin poisoning

Prognostic factors in insulin poisoning are subject to debate. It is generally accepted that the severity of intoxication should be assessed based on clinical findings rather than on any speculated amount of self-injected insulin [1,13]. The interval between insulin self-injection and initiation of therapy (>10 hours) and the duration of the hypoglycaemic coma were proposed to be relevant prognostic factors [13,14]. Our findings were consistent with the reported literature in that we identified two independent outcome predictors: delayed initiation of dextrose infusion (>6 hours) and duration of mechanical ventilation (>48 hour; a surrogate marker of the severity of the hypoglycaemic encephalopathy). Interestingly, as in our study, the dose and type of insulin were found to be closely related to the duration but not to the severity of hypoglycaemia [1,13,15]. It should be noted that patients may become hypoglycaemic much later than predicted based on the conventional duration of action of insulin preparations [7].

The cause of the dissociation between large doses of insulin and the severity of subsequent hypoglycaemia remains unclear [16]. In addition to activation of counter-regulatory mechanisms, a rate-limiting system appears to be involved in the blood glucose response to plasma insulin level, which is not affected by increased circulating insulin [16,17]. This is supported by the comparable efficacy between low-dose and conventional high-dose insulin therapy in diabetic ketosis [18]. Moreover, in diabetic patients who are chronically exposed to high levels of insulin, saturation of or decreased insulin receptors alters the response of blood glucose to circulating insulin [16]. It has also been hypothesized that there is a delayed dissociation of insulin bound to antibodies *in vivo*, but this is considered rather unlikely [16]. In contrast, duration of hypoglycaemia is usually much longer than predicted based on the commonly accepted kinetics of insulin absorption and action, whereas the degree of hypoglycaemia may not be so profound, especially in patients who have diabetes [7]. Some diabetic patients have defects in counter-regulatory hormone secretion, resulting in impaired recovery from insulin-induced hypoglycaemia [19]. In other cases, hypoglycaemia induces a reduction in peripheral circulation, limiting the absorption of the subcutaneously self-injected insulin [7].

### Insulin kinetics in acute intoxication

Study of the kinetics of self-injected insulin is difficult, particularly in nondiabetic patients, because of the presence of endogenous insulin. Thus, in order to interpret accurately the insulin levels and to study the disappearance of exogenous insulin from circulation, we used the level of peptide C (a cleavage product of endogenous pro-insulin) as a surrogate, the value of which has previously been demonstrated [6,20]. We considered the existence of suppressed C-peptide immunoreactivity and a molar ratio of insulin to C-peptide of less than 1 (unity) to represent assurance of reliable measurement of exogenous insulin [21].

The kinetics of insulin follow a multi-compartmental course, with a terminal plasma half-life of 10 to 20 minutes [22]. Insulin metabolism is dependent on hepatic and renal functions, with a small contribution made by muscle and adipose tissue [14]. Using a non-compartmental analysis in a case of insulin intoxication in a type 1 diabetic patient, Shibutani and Ogawa [17] found an elimination half-life of 6.2 hours. In another insulin-poisoned type 1 diabetic patient, Fasching and coworkers [23] identified a biphasic slow decline, with apparent half-lives of 4 hours and 10 hours for the two successive phases, respectively. In our patients, we identified late half-lives ranging from 0.8 to 11.7 hours, depending on the insulin type.

The kinetics of insulin are characterized by a large intra-individuals and inter-individual variability [13]. In acute poisoning, insulin levels reflect various delays in insulin activity, including delayed absorption from the injection site and possibly prolonged clearance of absorbed insulin. Compared with usual use of insulin, which is completely absorbed within 24 hours, time to peak concentration is delayed, suggesting slow absorption from the injected site. Several factors may alter insulin kinetics, resulting in prolonged elimination and consequently prolonged duration of action. Large volumes of self-injected insulin solution may cause a 'depot effect', resulting in a significant reduction in local blood flow caused by compression of tissues at the injection site. In diabetic patients, absorption is also delayed if local lipodystrophy caused by repeated injections is present. Circulating antibodies against insulin as well as impaired renal and hepatic function may also alter insulin clearance.

### Insulin toxicokinetic/toxicodynamic relationships in acute intoxications

TK/TD relationships allow descriptive and quantitative characterization of the time course of in *vivo *drug effect in relation to its corresponding drug concentration within an individual [24]. To our knowledge, there is no case of human insulin poisoning with a TK/TD analysis addressing the effects of insulin on glycaemia. We used the glucose infusion rate as a surrogate marker of the severity of hypoglycaemia. In the six patients the maximal glucose infusion rate was associated with a wide range of insulin concentrations, suggesting a saturable toxic mechanism at these high concentrations. Consistent with this, insulin-stimulated glucose flux is a saturable, receptor-mediated process with a nonlinear dose-effect curve [25,26]. The range of insulin concentrations accompanied by a decrease in glucose infusion rate was highly variable, enhancing the weak prognostic value of circulating insulin concentration. In contrast, the rate of glucose infusion decreased only when plasma insulin concentrations dropped dramatically. Our findings clearly demonstrate that prompt recognition and adequate treatment of the hypoglycaemic events is the key to achieving a successful outcome.

Whether there is any correlation between amounts of administered glucose and self-administered insulin is subject to debate [7,13]. As stated above, insulin level is not related to the severity of hypoglycaemia. Insulin lowers serum glucose levels by increasing the glucose uptake of insulin-sensitive cells, stimulating oxidative and nonoxidative glucose metabolism and suppressing hepatic glycogenolysis and gluconeogenesis. At plasma insulin concentrations of 50 to 60 μU/ml, complete inhibition of liver glucose production occurs [27]. Because hepatic glucose output is completely suppressed at high insulin concentration [28,29], glucose requirement is entirely met by exogenous glucose. Glucose infusion represents the only guarantee of safe outcome in severe poisonings. In the presence of extremely high plasma insulin concentrations, as occur in overdose, glucose dynamics closely resemble those observed in healthy nondiabetic patients and type 1 diabetes during euglycaemic hyperinsulinaemic clamp (10 mU/kg per minute) [23,25]. When serum insulin levels fall below the level necessary to suppress hepatic glucose production, exogenous requirements decrease and hypoglycaemia subsides.

The cornerstone of the treatment of insulin poisoning remains continuous glucose repletion to avoid ongoing or recurrent hypoglycaemia, coupled with frequent glucose monitoring [6,14]. In addition, the efficiency of glucagon in insulin overdose is controversial and is dependent on hepatic glycogen stores, which are likely to be quickly exhausted in insulin-poisoned patients. Basal glucose utilization (2 mg/kg per minute) at postprandial physiological insulin concentrations up to 719 pmol/l [26] may increase 3–6 fold in the presence of high circulating concentrations of insulin of up to 1000 μU/ml [6,7,30]. The maximal glucose disposal rate of 400 mg/m^2 ^per minute (10 to 12 mg/kg per minute) was determined in normal volunteers using the euglycaemic hyperinsulinaemia glucose clamp technique [28,31,32]. Thus, in severe insulin poisoning the anticipated maximum glucose requirement should be 6 to 12 mg/kg per minute [7]. In patient 3 we observed unusually elevated rates of glucose infusion (E_max _23 mg/kg per minute). Thus, we believe that, in this case, co-ingestion of other antidiabetic medications (1,700 mg metformin, 10 mg glibenclamid, and 100 mg acarbose) enhanced insulin-related glucose requirements.

Because insulin kinetics are linear using logarithmic transformation, Shibutani and Ogawa [17] suggested that duration of the subsequent hypoglycaemia and the required glucose administration could easily be determined. Relationships between the amount of self-injected insulin and the total amount of intravenous glucose administered or the total time of intravenous glucose treatment were determined [7]. However, as clearly demonstrated in our patients, the optimal glucose infusion is difficult to determine because of the delayed and erratic absorption of the injected insulin, varying kinetics (especially when different types of insulin were injected) and the likelihood of both immediate and recurrent hypoglycaemia in nondiabetic as compared with diabetic patients [6]. Kinetics of maximal glucose use in diabetic or obese patients is markedly different from those in normal individuals because of post-receptor abnormalities or downregulation of insulin receptors as a consequence of the hyperinsulinaemia associated with over-eating [6]. Moreover, people without diabetes are more likely than diabetic patients to develop recurrent hypoglycaemia, in relation to the lack of insulin antibodies, insulin resistance and endogenous insulin secretion, in response to glucose infusion [1,7,33]. Thus, because fixed and excessive glucose load may induce significant metabolic complications, including hepatic steatosis and lactic acidosis [34], treatment should be based on titrated continuous glucose infusion with additional boluses when necessary to maintain glucose levels in the range 10 to 12 mmol/l.

### Study limitations

Our study has several limitations. We present insulin poisoning outcomes from just one centre. The number of patients might have been insufficient to yield any persuasive, statistically significant findings. Definitive conclusions regarding the diagnostic value of plasma insulin concentrations should thus be drawn with caution. Moreover, the kinetic study was performed in two nondiabetic patients, using only three time points for which data regarding insulin concentrations with corresponding suppressed C-peptide levels were available, to be sure that we only considered exogenous insulin. Finally, the variability of circumstances, the duration of action of the injected insulin, the underlying morbidities and the co-ingested medications may preclude drawing of any definitive conclusions regarding the amount of glucose required to correct hypoglycaemia and the supposed injected insulin doses or plasma concentrations.

## Conclusion

Insulin self-overdoses are rare. However, they may have severe neurological sequelae and result in death. Assessment of prognosis relies on clinical findings. The plasma EC_50 _is about 46 mIU/l. TK/TD relationships are helpful in quantifying the need for glucose repletion. However, because of the difficulty in obtaining insulin concentration measurements and the marked inter-individual variability in response to insulin, careful monitoring of serum glucose level and accordingly adjusted glucose infusion remain key to optimizing prognosis after poisoning.

## Key messages

• Although rare, insulin self-overdose may have severe neurological sequelae and result in death.

• Careful monitoring of serum glucose level and adjusted glucose infusion rate are key to optimizing prognosis after insulin poisoning.

• A delay between insulin injection and first medical treatment in excess of 6 hours and a duration of mechanical ventilation in excess of 48 hours are significant, independent predictors of unfavourable outcome after insulin poisoning.

• During the course of insulin poisoning, TK/TD relationships between the glucose infusion rate (E) and insulin concentrations (C) fit the E_max _model E = (E_max _× C)/(EC_50 _+ C).

• The plasma EC_50 _is about 46 mIU/l.

• In insulin self-overdose, kinetics of exogenous insulin are of the first order, resulting in a linear decrease in concentrations using a semi-logarithmic scale.

## Abbreviations

CI = confidence interval; CPC = Cerebral Performance Category; E_max _= maximum measured glucose infusion rate; EC_50 _= insulin concentration associated with the half-maximum glucose infusion rate; ICU = intensive care unit; OR = odds ratio; SAPS = Simplified Acute Physiology Score; TK/TD = toxicokinetic/toxicodynamic.

## Competing interests

The authors declare that they have no competing interests.

## Authors' contributions

BM designed the study, wrote the protocol, collected data, carried out analyses and wrote the manuscript. ND performed statistical analysis. VB analyzed data and performed TK/TD modelling. RS helped to review the radiological findings. CC performed the insulin assay. JML performed the insulin assay and participated in the study design. FJB conceived and coordinated the study. All authors read and approved the final manuscript.

## References

[B1] Spiller HA (1998). Management of antidiabetic medications in overdose. Drug Saf.

[B2] Lai MW, Klein-Schwartz W, Rodgers GC, Abrams JY, Haber DA, Bronstein AC, Wruk KM (2006). 2005 Annual Report of the American Association of Poison Control Centers' national poisoning and exposure database. Clin Toxicol (Phila).

[B3] von Mach MA, Meyer S, Omogbehin B, Kann PH, Weilemann LS (2004). Epidemiological assessment of 160 cases of insulin overdose recorded in a regional poisons unit. Int J Clin Pharmacol Ther.

[B4] Jefferys DB, Volans GN (1983). Self poisoning in diabetic patients. Hum Toxicol.

[B5] Lionte C, Sorodoc L, Laba V (2004). Toxic-induced hypoglycaemia in clinical practice. Rom J Intern Med.

[B6] Roberge RJ, Martin TG, Delbridge TR (1993). Intentional massive insulin overdose: recognition and management. Ann Emerg Med.

[B7] Stapczynski JS, Haskell RJ (1984). Duration of hypoglycemia and need for intravenous glucose following intentional overdoses of insulin. Ann Emerg Med.

[B8] Le Gall JR, Lemeshow S, Saulnier F (1993). A new Simplified Acute Physiology Score (SAPS II) based on a European/North American multicenter study. JAMA.

[B9] Jennett B, Bond M (1975). Assessment of outcome after severe brain damage: a practical scale. Lancet.

[B10] Pearson MJ, Martin FI (1970). The separation of total plasma insulin from binding proteins using gel filtration: its application to the measurement of rate of insulin disappearance. Diabetologia.

[B11] von Mach MA, Gauer M, Meyer S, Omogbehin B, Schinzel H, Kann PH, Weilemann LS (2006). Antidiabetic medications in overdose: a comparison of the inquiries made to a regional poisons unit regarding original sulfonylureas, biguanides and insulin. Int J Clin Pharmacol Ther.

[B12] Yanagawa Y, Isoi N, Tokumaru AM, Sakamoto T, Okada Y (2006). Diffusion-weighted MRI predicts prognosis in severe hypoglycemic encephalopathy. J Clin Neurosci.

[B13] Arem R, Zoghbi W (1985). Insulin overdose in eight patients: insulin pharmacokinetics and review of the literature. Medicine (Baltimore).

[B14] Moore DF, Wood DF, Volans GN (1993). Features, prevention and management of acute overdose due to antidiabetic drugs. Drug Saf.

[B15] Samuels MH, Eckel RH (1989). Massive insulin overdose: detailed studies of free insulin levels and glucose requirements. J Toxicol Clin Toxicol.

[B16] Martin FI, Hansen N, Warne GL (1977). Attempted suicide by insulin overdose in insulin-requiring diabetics. Med J Aust.

[B17] Shibutani Y, Ogawa C (2000). Suicidal insulin overdose in a type 1 diabetic patient: relation of serum insulin concentrations to the duration of hypoglycemia. J Diabetes Complications.

[B18] Page MM, Alberti KG, Greenwood R, Gumaa KA, Hockaday TD, Lowy C, Nabarro JD, Pyke DA, Sonksen PH, Watkins PJ, West TE (1974). Treatment of diabetic coma with continuous low-dose infusion of insulin. BMJ.

[B19] Bolli GB, Dimitriadis GD, Pehling GB, Baker BA, Haymond MW, Cryer PE, Gerich JE (1984). Abnormal glucose counterregulation after subcutaneous insulin in insulin-dependent diabetes mellitus. N Engl J Med.

[B20] Bauman WA, Yalow RS (1984). Hyperinsulinemic hypoglycemia. Differential diagnosis by determination of the species of circulating insulin. JAMA.

[B21] Lebowitz MR, Blumenthal SA (1993). The molar ratio of insulin to C-peptide. An aid to the diagnosis of hypoglycemia due to surreptitious (or inadvertent) insulin administration. Arch Intern Med.

[B22] Samuels MH, Eckel RH (1989). Massive insulin overdose: detailed studies of free insulin levels and glucose requirements. J Toxicol Clin Toxicol.

[B23] Fasching P, Roden M, Stuhlinger HG, Kurzemann S, Zeiner A, Waldhausl W, Laggner AN (1994). Estimated glucose requirement following massive insulin overdose in a patient with type 1 diabetes. Diabet Med.

[B24] Baud FJ (1998). Pharmacokinetic-pharmacodynamic relationships. How are they useful in human toxicology?. Toxicol Lett.

[B25] Fasching P, Ratheiser K, Damjancic P, Schneider B, Nowotny P, Vierhapper H, Waldhausl W (1993). Both acute and chronic near-normoglycaemia are required to improve insulin resistance in type 1 (insulin-dependent) diabetes mellitus. Diabetologia.

[B26] DeFronzo RA, Ferrannini E (1982). Influence of plasma glucose and insulin concentration on plasma glucose clearance in man. Diabetes.

[B27] Butler PC, Rizza RA (1989). Regulation of carbohydrate metabolism and response to hypoglycemia. Endocrinol Metab Clin North Am.

[B28] Olefsky JM, Kolterman OG (1981). Mechanisms of insulin resistance in obesity and noninsulin-dependent (type II) diabetes. Am J Med.

[B29] Revers RR, Kolterman OG, Scarlett JA, Gray RS, Olefsky JM (1984). Lack of in vivo insulin resistance in controlled insulin-dependent, type I, diabetic patients. J Clin Endocrinol Metab.

[B30] Christensen NJ, Orskov H (1968). The relationship between endogenous serum insulin concentration and glucose uptake in the forearm muscles of nondiabetics. J Clin Invest.

[B31] DeFronzo RA, Tobin JD, Andres R (1979). Glucose clamp technique: a method for quantifying insulin secretion and resistance. Am J Physiol.

[B32] Ludvik B, Nolan JJ, Roberts A, Baloga J, Joyce M, Bell JM, Olefsky JM (1995). A noninvasive method to measure splanchnic glucose uptake after oral glucose administration. J Clin Invest.

[B33] Bayly GR, Ferner RE (1993). Persistent insulin secretion after insulin overdose in a non-diabetic patient. Lancet.

[B34] Jolliet P, Leverve X, Pichard C (2001). Acute hepatic steatosis complicating massive insulin overdose and excessive glucose administration. Intensive Care Med.

